# Characterization of the proteins encoded by a recently emerged cotton-infecting *Polerovirus*

**DOI:** 10.1007/s11262-024-02086-3

**Published:** 2024-06-21

**Authors:** Mary F. Akinyuwa, Bailee K. Price, Sung-Hwan Kang

**Affiliations:** 1 Department of Entomology and Plant Pathology, Auburn University, Auburn, AL 36849 USA; 2 College of Sciences and Mathematics, Auburn University, Auburn, AL 36849 USA; 3 Present Address: Corteva Agriscience, Indianapolis, IN 46268 USA; 4 Present Address: Whiddon College of Medicine, University of South Alabama, Mobile, AL 36688 USA

**Keywords:** CLDV, CLRDV, Reactive oxygen species, Intracellular localization, *Polerovirus*

## Abstract

The cotton leafroll dwarf virus (CLDV), an important viral pathogen responsible for substantial losses in cotton crops, has recently emerged in the United States (US). Although CLDV shares similarities with other members of the genus *Polerovirus* in terms of encoded proteins, their functional characteristics remain largely unexplored. In this study, we expressed and analyzed each protein encoded by CLDV to determine its intracellular localization using fluorescence protein fusion. We also evaluated their potential to induce plant responses, such as the induction of hypersensitive response-like necrosis and the generation of reactive oxygen species. Our findings show that the proteins encoded by CLDV exhibit comparable localization patterns and elicit similar robust plant responses as observed with cognate proteins from other viruses within the genus *Polerovirus*. This study contributes to our understanding of the functional repertoire of genes carried by *Polerovirus* members, particularly to CLDV that has recently emerged as a widespread viral pathogen infecting cotton in the US.

**Supplementary Information:**

The online version contains supplementary material available at 10.1007/s11262-024-02086-3.

Recently, viral disease symptoms were observed in cotton plants within the United States (US) cotton belt [1]. Sequencing analysis of the RNA genome isolated from symptomatic cotton plants revealed the presence of a viral genome closely related to cotton leafroll dwarf virus (CLDV, commonly known as CLRDV), a species previously reported in South America that has caused substantial yield losses by inducing cotton blue disease [1–3]. CLDV belongs to the genus *Polerovirus*, family *Solemoviridae* [4], which comprises plant viruses expressing their proteins through complex translation strategies from a positive-sense, single-stranded RNA genome approximately 5.8 kb in length [1, 2, 5]. The CLDV genome is organized into seven open reading frames (ORFs), akin to other members of the genus *Polerovirus* (see Supplementary Fig. 1). The translation mechanism and the function of proteins encoded by each ORF have been studied extensively in other poleroviruses (reviewed in [6]). ORF 0 encodes the P0 protein, which functions as a viral suppressor of RNA silencing (VSR) to counteract the RNA silencing mechanism in plants [7–15]. ORF 1 encodes the P1 protein, which is involved in viral RNA replication and genomic RNA synthesis, along with a P1–P2 fusion protein translated by a—1 ribosomal frameshift near the end of ORF 1 [16]. ORF 3a translates into the P3a protein via a non-canonical start codon, facilitating viral movement [17, 18]. ORF 3 and ORF 4 overlap, and the translation of P3 and P4 is determined by leaky scanning. P3 functions as a coat protein (CP), while P4 serves as a movement protein (MP) [19–23]. ORF 3-ORF 5 produces a read-through protein (P3-5), which is essential for aphid transmission and virus movement in plants [24–26].

**Fig. 1:**
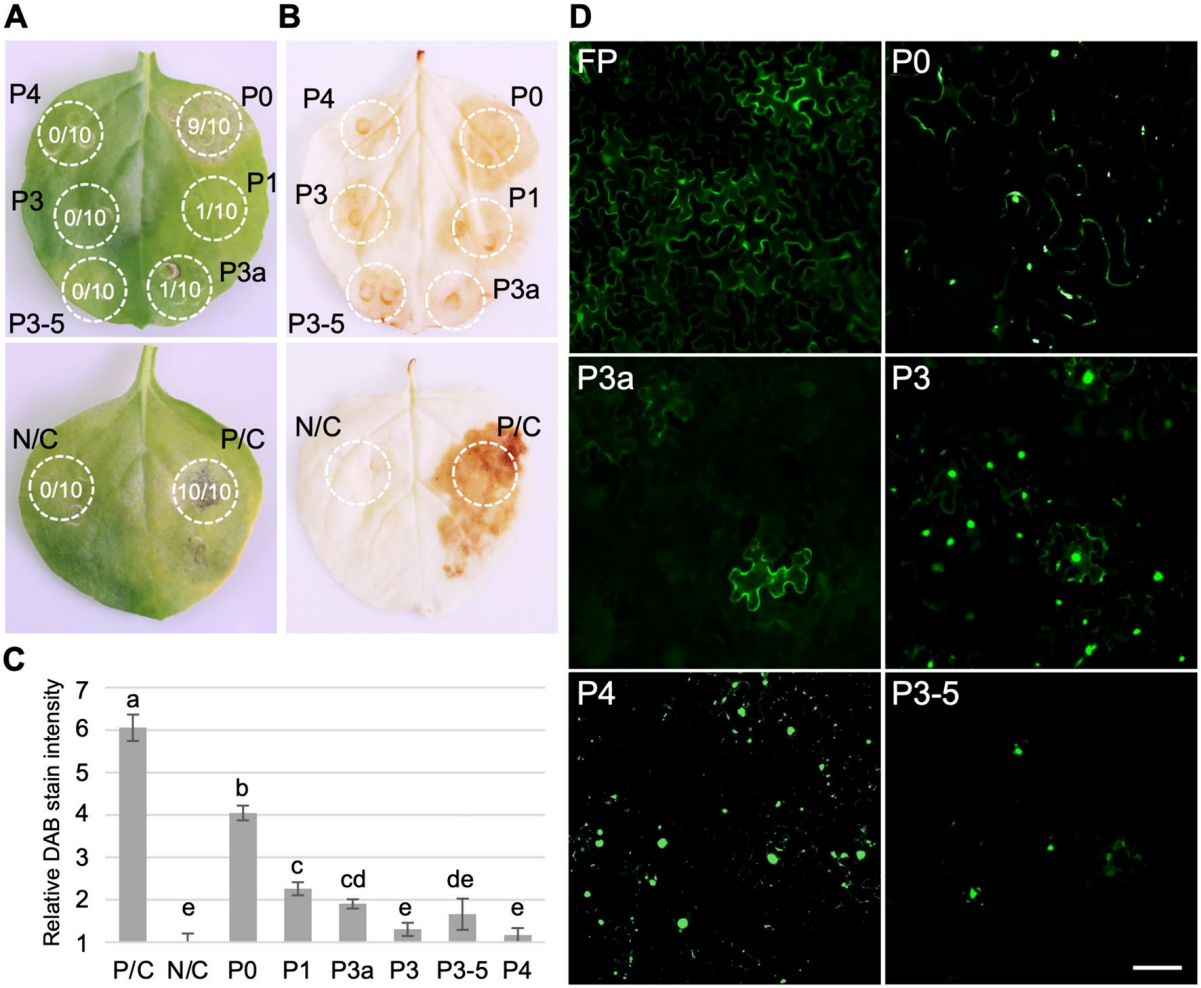
Hypersensitive response (HR)-like necrosis induction and intracellular localization of CLDV-encoded proteins. Six-week-old *N. benthamiana* plants were infiltrated with *A. tumefaciens* GV3101 cells transformed with binary plasmid clones harboring CLDV ORFs. **A**bright-field images show necrotic phenotype within the infiltrated patches (white dotted circle). The number in the dotted circle indicates the ratio of patches showing necrotic phenotype. Images were taken at 8 dpi. P/C; turnip crinkle virus P38, N/C; empty vector. **B** *N. benthamiana* leaves were treated with 3,3ʹ.-diaminobenzidine (DAB) staining to demonstrate reactive oxygen species (ROS) accumulation within the infiltrated patches (white dotted circle). **C** The relative ROS production corresponding to the leaf patches expressing six CLDV proteins was analyzed by measuring the color intensity of DAB staining using ImageJ. Values are means from at least three independent patches per treatment. Error bars are standard deviation. Statistically significant differences, *p* < 0.01, determined by one-way ANOVA are denoted by letters. **D** Six-week-old *N. benthamiana* plants were infiltrated with *A. tumefaciens* GV3101 cells transformed with binary plasmid clones harboring fluorescence protein (FP)-tagged CLDV genes or a free FP. Images were taken using an epifluorescence microscope, echo revolve, at 4 dpi. Scale bar = 20 µm

Although CLDV presents a potential threat to the profitable production of economically important cotton crops, research into the functions of CLDV-encoded proteins has been limited, except for the P0 protein, which has been studied in the context of VSR [13–15, 27, 28]. Therefore, a more comprehensive investigation into CLDV-encoded proteins is warranted to understand their pathogenicity better and facilitate successful pest management for cotton diseases in future. As an initial step toward this goal, our study examined the molecular and cellular characteristics of the proteins produced by CLDV. Using two series of clones containing individual CLDV ORFs, we conducted experiments to assess hypersensitive response (HR)-like lesion elicitation and intracellular localization of CLDV-encoded proteins.

The induction of an HR-like response and the associated accumulation of reactive oxygen species (ROS) represent a fundamental plant basal defense response against viruses. During the early stages of pathogen invasion, including viral infections, the rapid buildup of ROS can trigger an HR-like response, resulting in lesions at the site of pathogen entry [29–31]. In interactions between plant hosts and viruses, the speed and intensity of the host’s immune response at the viral infection site often dictate the infection’s outcome. Therefore, viral proteins that contribute to generating HR-like lesions are considered primary factors in pathogenicity. The CLDV-encoded proteins responsible for triggering ROS accumulation and HR-like lesions were identified by visually examining *Nicotiana benthamiana* plants expressing each CLDV protein (Fig. 1A). Full-length genes of each ORF, amplified by polymerase chain reaction (PCR), were cloned into the binary plasmid pAIDEE (pAI [32]), which contains an upstream CaMV 35S promoter sequence (see Supplementary Table 1 for additional details regarding the constructs). The resulting constructs were used to transform *Agrobacterium tumefaciens* strain GV3101 for expressing the CLDV proteins in plants. Six CLDV proteins, P0, P1, P3a, P3, P4, and P3-5, were transiently expressed in six-week-old *N. benthamiana* via agroinfiltration of the transformed cells resuspended in buffer (10 mM MES, pH 5.85; 10 mM MgCl_2_; 150 µM acetosyringone) at the optical density 1.0 at 600 nm. The infiltrated plants were then maintained under a 16 h photoperiod for up to 10 days for the evaluation.

Within three days post-infiltration (dpi), leaf patches expressing the P0 protein or the P38 protein of turnip crinkle virus (P/C), which belongs to the genus *Betacarmovirus*, family *Tombusviridae* and has been documented to trigger a robust programmed cell death response [33], began to exhibit HR-like necrotic lesions (Fig. 1A). This evaluation continued up to 8 dpi, during which, among the CLDV-encoded proteins, only the patches expressing the P0 protein consistently produced HR-like lesions (Fig. 1A; shown by the number within white dotted circles). Furthermore, at 8 dpi, the severity of the necrotic symptoms caused by the P0 protein was less pronounced compared to those caused by the P/C. To explore whether the phenotype associated with the HR-like necrotic lesion formation was linked to ROS accumulation, we collected agroinfiltrated leaves at 3 dpi and subjected them to treatment with 3,3ʹ-diaminobenzidine (DAB) to detect hydrogen peroxide (Fig. 1B). For DAB staining, the detached leaves were washed three times with double distilled water and incubated overnight in 1 mg/ml DAB-HCl prepared in boric acid buffer (50 mM, pH 7.6). The leaves were subsequently incubated in 95% ethanol with three changes before the images were taken. The staining revealed brown pigmentation in all constructs within the infiltrated patches (Fig. 1B; depicted by white dotted circles). Notably, the intensity of the brown pigmentation was visibly stronger in patches infiltrated with the P0 or P/C compared to those infiltrated with other CLDV proteins. To better assess the relative ROS accumulation levels, we quantified the intensity of the DAB staining by measuring the pixel intensity of the infiltrated patches using an ImageJ software [34] (Fig. 1C). The analysis indicated that the P0 protein triggered significantly more ROS accumulation than the other CLDV proteins (Fig. 1C; denoted as ‘b’). As anticipated from the visual lesion evaluation, the level of ROS accumulation induced by the P0 protein was significantly lower than that caused by the P/C (Fig. 1C; denoted as ‘a’). These results suggest that among the proteins produced by CLDV, only the P0 protein induces an HR-like response and ROS accumulation, reinforcing its potential role as a pathogenicity factor. It is worth noting that P0 proteins encoded by other poleroviruses have also been shown to similarly trigger HR-like lesions and ROS accumulation in viruses such as sugarcane yellow leaf virus, turnip yellows virus (TuYV), potato leafroll virus (PLRV), brassica yellows virus, and pepper vein yellows virus [35–38]. However, these studies involving other poleroviruses have not reported the effects of other proteins encoded by them. To the best of the author’s knowledge, this is the first study to report such a comprehensive survey encompassing multiple poleroviral proteins.

Understanding the intracellular localization of virus-encoded proteins within the host provides insight into their functions and roles in the virus infection. Previous studies on the subcellular localization of poleroviral proteins have elucidated some of their key mechanisms during host infection by TuYV [10, 17] and PLRV [39–41]. To extend our understanding of the proteins produced by *Polerovirus*, we examined the intracellular localization of CLDV proteins by expressing each of them fused to the green fluorescent protein (FP) ORF. The cDNA of FP-tagged CLDV ORFs was placed under the CaMV 35S promoter sequence in the pAI plasmid (see Supplementary Table 1 for additional details regarding the constructs) and introduced into *A. tumefaciens* GV3101 for ectopic expression by agroinfiltration in *N. benthamiana* plants. At 4 dpi, the mesophyll cells of the infiltrated leaves were observed using a fluorescence microscope with either FITC cube (EX:470 ± 40 and EM:525 ± 50) or TxRED cube (EX:560 ± 40 and EM:630 ± 75) for the fluorescence detection (Fig. 1D).

All five CLDV protein-tagged FPs showed localization patterns different from the control FP, to which no CLDV protein was tagged (Fig. 1D; FP). Strong nuclear fluorescence was observed from the CLDV P3-tagged FP (Fig. 1D; P3), similar to what has been shown to be mediated by nuclear localization signal of PLRV P3 [39]. The CLDV P4-tagged FP displayed multiple fluorescent speckles along the membrane (Fig. 1D; P4). The observed speckles resemble typical plasmodesmata localization previously shown for PLRV P4 [40]. Some fluorescence was observed from the CLDV P3-5 RTP-tagged FP mainly in the nucleus (Fig. 1D; P3-5). The fluorescence of CLDV P3a-tagged FP was mainly found along the membrane without any trace in the nucleus (Fig. 1D: P3a), suggesting subcellular localization similar to P3a encoded by other poleroviruses. Indeed, CLDV P3a protein has a putative transmembrane domain [17]. As previously reported [27], the CLDV P0-tagged FP exhibited significant fluorescence appearing as multiple speckles distributed along the membrane with some observed in the nucleus (Fig. 1D: P0). Although the intracellular localization of CLDV proteins seems to be very similar to cognate proteins from other poleroviruses, further investigation is needed to better understand the role of each CLDV protein during viral infection.

Overall, the surveyed characteristics of CLDV-encoded proteins were comparable to their cognate proteins produced by other viruses within the genus *Poleovirus*. Further investigation into the underlying mechanisms of their cellular location within the primary host, cotton, could enhance our understanding of their biological functions and their roles in virus pathogenesis. Such insights could pave the way for developing effective strategies by specifying the targets to consider for the development of genetically modified cotton or selecting them for breeding programs to protect cotton crops from virus infections, thereby promoting sustainable CLDV management in cotton crops.

## Supplementary Information

Below is the link to the electronic supplementary material.

Supplementary material 1 ( TIFF 5,102 kb) Schematic representation of the CLDV genome. Overlapping open reading frames (ORFs) are represented as gray arrowhead boxes. UTR; untranslated region. This figure was adapted from Akinyuwa and Kang (2024)

Supplementary material 2 (DOCX 28)

## Data Availability

The data generated or analyzed during this study are included in this published article.
