# Supplementary figures and images for: Characterization of the proteins encoded by a recently emerged cotton-infecting Polerovirus
Source: Virus Genes. 2024 Jun 21;60(5):563–7. doi: 10.1007/s11262-024-02086-3 (PMC11384633; doi:10.1007/s11262-024-02086-3)

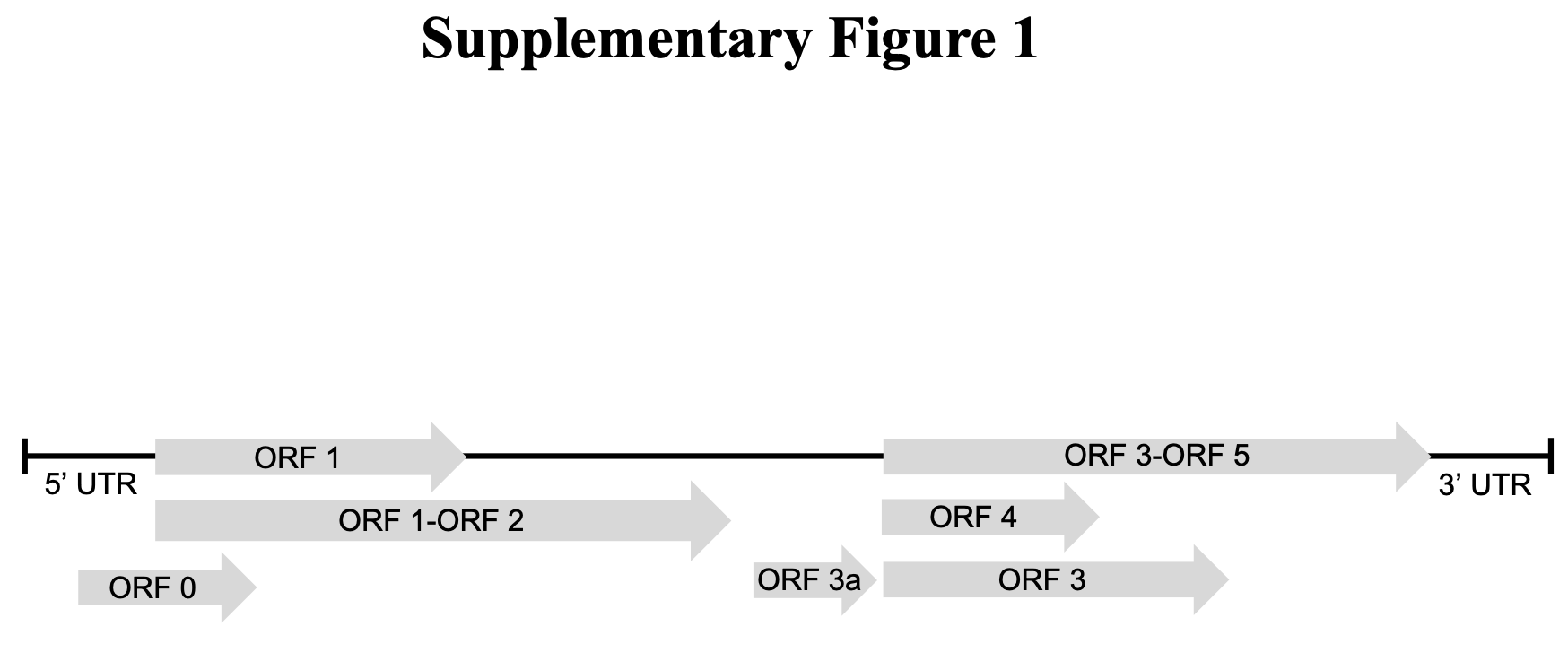

Supplement: Supplementary file 1 — Supplementary material 1 ( TIFF 5,102 kb) Schematic representation of the CLDV genome. Overlapping open reading frames (ORFs) are represented as gray arrowhead boxes. UTR; untranslated region. This figure was adapted from Akinyuwa and Kang (2024) [file 11262_2024_2086_MOESM1_ESM.tiff]
