# Supplementary material for: Characterization of the proteins encoded by a recently emerged cotton-infecting Polerovirus
Source: Virus Genes. 2024 Jun 21;60(5):563–7. doi: 10.1007/s11262-024-02086-3 (PMC11384633; doi:10.1007/s11262-024-02086-3)
Supplement: Supplementary file 2 — Supplementary material 2 (DOCX 28) [file 11262_2024_2086_MOESM2_ESM.docx]

**Supplementary Table 1.** List of oligomers used for the generation of constructs.

| Name​ | Sequence ​(5′ to 3′) | References^3^ |
| --- | --- | --- |
| Non-tagged CLDV constructs (Figures 1A and 1B)^1^ | | |
| P0 FW ApaI | ACTA*GGGCCC*AACAATGTTGAATTTGATCATCTGC | [27] |
| P0 RV XbaI | GGAC*TCTAGA*TCAACTGCTTTCTCCTTCAC | [27] |
| P1 FW ApaI | ACTA*GGGCCC*AACAATGTCTTTAATGAGTCTTTTC |  |
| P1 RV XbaI | GTAA*TCTAGA*TCAGCTCCGCTTGGGGGCCG |  |
| P3 FW ApaI | ACTA*GGGCCC*AACAATGAATACGGTCGTGGGTAG |  |
| P3 RV XbaI | GTAC*TCTAGA*CTATTTGGGGTTATGGAATTG |  |
| P3a FW ApaI | ACTA*GGGCCC*AACAATGCTGGACTACAAATTCCTATCAG |  |
| P3a RV XabI | GTAC*TCTAGA*CTACCCACGACCGTATTCATTAAC |  |
| P4 FW ApaI | ACTA*GGGCCC*AACAATGGAAGAAGACGACCACGTAG |  |
| P4 RV XbaI | GTAC*TCTAGA*CTATCGACGAGGAACCATTGC |  |
| P3-5 FW ApaI | ACTA*GGGCCC*AACAATGAATACGGTCGTGGGTAG |  |
| P3-5 RV XabI | GTAC*TCTAGA*TTACCTATCCATCACCTTTTGC |  |
| CLDV protein-tagged FP constructs (Figures 1D)^2^ | | |
| P0 FP FW | TATGTGAAGGAGAAAGCAGTATGGCTAGCAAAGGAGAAGA | [27] |
| P0 FP RV | TCTTCTCCTTTGCTAGCCATACTGCTTTCTCCTTCACATA | [27] |
| P3 FP FW | GCCAATTCCATAACCCCAAAATGGCTAGCAAAGGAGAAGA |  |
| P3 FP RV | TCTTCTCCTTTGCTAGCCATTTTGGGGTTATGGAATTGGC |  |
| P3a FP FW | TTAATGAATACGGTCGTGGGATGGCTAGCAAAGGAGAAGA |  |
| P3a FP RV | TCTTCTCCTTTGCTAGCCATCCCACGACCGTATTCATTAA |  |
| P4 FP FW | AGGCAATGGTTCCTCGTCGAATGGTGTCTAAGGGCGAAGA |  |
| P4 FP RV | TCTTCGCCCTTAGACACCATTCGACGAGGAACCATTGCCT |  |
| P3-5 FP FW | GCCAATTCCATAACCCCAAAATGGCTAGCAAAGGAGAAGA |  |
| P3-5 FP RV | TCTTCTCCTTTGCTAGCCATTTTGGGGTTATGGAATTGGC |  |
| FP RV XbaI | GTAC*TCTAGA*CTATTTGTAGAGCTCATCC | [27] |
| Sequence verification | | |
| pAI SEQ FW | CCTCGAGAATTCTCAACACAAC | [15] |
| pAI SEQ RV | GCTCAACACATGAGCGAAACCC | [15] |

^1^ The full-length genes of each ORF were amplified by PCR using a pair of primers (column 1; Name), one with *Apa*I restriction endonuclease recognition site at its 5’ end and the other with *Xba*I restriction endonuclease recognition site at its 3’ end. Restriction endonuclease (*Xba*I and *Apa*I) recognition sequences are *italicized and underlined* (column 2; Sequence). The amplified DNAs were digested using *Apa*I and *Xba*I restriction endonucleases and then ligated into the corresponding region of the binary plasmid pAI digested with the same restriction endonucleases using T4 DNA ligase. *E. coli* NEB^®^ DH 5-alpha cells were transformed with the constructs and screened on LB agar supplemented with kanamycin. Final products were verified by Sanger sequencing using the primers listed above.

^2^ The CLDV protein-tagged FPs were generated by overlap-extension PCR using two PCR-generated fragments. First fragments were amplified using the forward primers for the non-tagged CLDV constructs and the reverse primers listed here for each gene. Second fragments were amplified using the forward primers and the FP RV XbaI listed here for each gene. Two amplified DNA fragments were purified and used as templates for a subsequent PCR using the forward primers for the non-tagged CLDV constructs and the FP RV XbaI. The final amplified DNAs were then processed for the ligation, screening, and sequencing as described above. Restriction endonuclease (*Xba*I) recognition sequence is *italicized and underlined* (column 2; Sequence).

^3^ Previously published oligomers were noted. Otherwise, designed in this study.
